# Sleep deprivation leads to a loss of functional connectivity in frontal brain regions

**DOI:** 10.1186/1471-2202-15-88

**Published:** 2014-07-19

**Authors:** Ilse M Verweij, Nico Romeijn, Dirk JA Smit, Giovanni Piantoni, Eus JW Van Someren, Ysbrand D van der Werf

**Affiliations:** Netherlands Institute for Neuroscience, an Institute of the Royal Netherlands Academy of Arts and Sciences, Meibergdreef 47, 1105 BA Amsterdam, the Netherlands; Department of Psychology, VU University, Amsterdam, the Netherlands; Department of Integrative Neurophysiology, Faculty of Earth and Life Sciences, VU University, Amsterdam, the Netherlands; Department of Medical Psychology, VU University Medical Centre, Amsterdam, the Netherlands; Department of Anatomy and Neurosciences, VU University Medical Centre, Amsterdam, the Netherlands

**Keywords:** Sleep deprivation, Brain connectivity, Graph theory, EEG analysis, Small-world networks

## Abstract

**Background:**

The restorative effect of sleep on waking brain activity remains poorly understood. Previous studies have compared overall neural network characteristics after normal sleep and sleep deprivation. To study whether sleep and sleep deprivation might differentially affect subsequent connectivity characteristics in different brain regions, we performed a within-subject study of resting state brain activity using the graph theory framework adapted for the individual electrode level.

In balanced order, we obtained high-density resting state electroencephalography (EEG) in 8 healthy participants, during a day following normal sleep and during a day following total sleep deprivation. We computed topographical maps of graph theoretical parameters describing local clustering and path length characteristics from functional connectivity matrices, based on synchronization likelihood, in five different frequency bands. A non-parametric permutation analysis with cluster correction for multiple comparisons was applied to assess significance of topographical changes in clustering coefficient and path length.

**Results:**

Significant changes in graph theoretical parameters were only found on the scalp overlying the prefrontal cortex, where the clustering coefficient (local integration) decreased in the alpha frequency band and the path length (global integration) increased in the theta frequency band. These changes occurred regardless, and independent of, changes in power due to the sleep deprivation procedure.

**Conclusions:**

The findings indicate that sleep deprivation most strongly affects the functional connectivity of prefrontal cortical areas. The findings extend those of previous studies, which showed sleep deprivation to predominantly affect functions mediated by the prefrontal cortex, such as working memory. Together, these findings suggest that the restorative effect of sleep is especially relevant for the maintenance of functional connectivity of prefrontal brain regions.

## Background

Functional connectivity between brain areas determines the way the brain processes information. Several studies [1–4] suggest that sleep is important for the activity and recruitment of different brain areas to form networks for optimal information processing during the wake state; yet the differential sensitivity of brain areas to the effects of sleep and conversely, sleep deprivation, have hardly been addressed. We here report findings of regionally specific and frequency-dependent effects of sleep deprivation on brain functional networks.

Functional MRI (fMRI) studies have shown that correlations between activity of frontal and posterior areas of the Default Mode Network (DMN), a resting state network that is suppressed during a task, are attenuated during deep sleep [5, 6]. Gujar et al. [1] showed that sleep loss triggers an imbalance in the activation of midline posterior and anterior brain regions of the DMN during subsequent wake. The magnitude of this imbalance was related to the amount of prior sleep of the subjects. Shao et al. [7] showed that connectivity with subcortical areas is also affected by sleep deprivation; functional connectivity between the thalamus and frontal and temporal gyri was decreased after sleep deprivation. This implies that sleep affects activity and (cortical and subcortical) functional connectivity between brain areas during rest and that specific brain areas show vulnerability to the effect of sleep deprivation.

**Figure 1:**
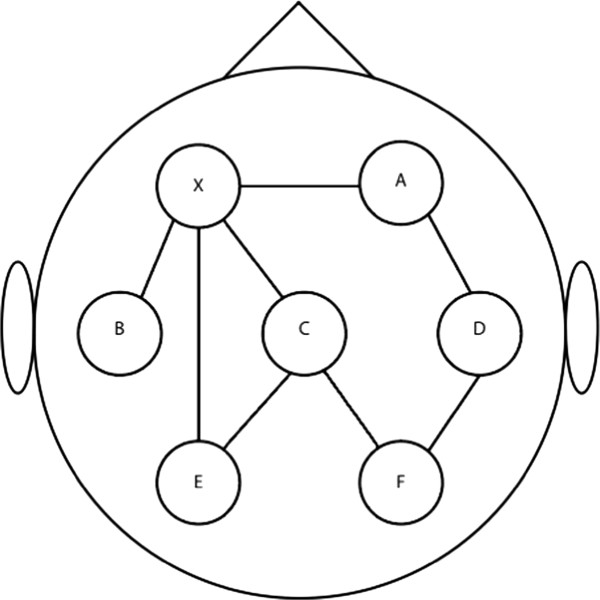
**Clustering coefficient and path length.** The circles represent brain areas (nodes) and the lines between the circles represent the connections between nodes (edges). It takes one step to go from node X to node A, two steps to go from node X to node D etc. The average path length of node X is therefore 1(A) + 1(B) + 1(C) + 2(D) + 3(E) + 2(F) /6 = 1.67. Node X is directly connected to node A, B and C (neighbors) the clustering coefficient (proportion of neighbors of node X that are also connected to each other) is therefore 2/3 = 0.67.

**Figure 2:**
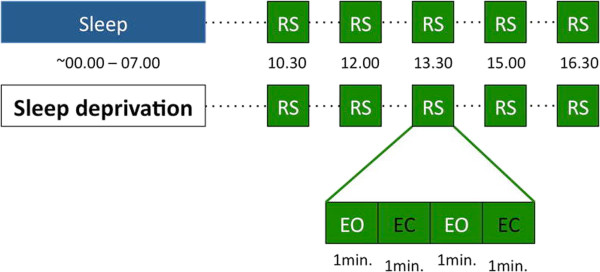
**Schematic depiction of the protocol.** After a night of normal sleep or total sleep deprivation (verified with actigraphy and sleep diary), subjects underwent a protocol of repeated resting-state (RS) measurements using high-density 61-channel EEG. Each RS block consisted of 4 alternating 1-minute eyes-open (EO) or eyes-closed (EC) measurements.

To capture network dynamics of brain functioning, Graph Theory offers an insightful framework. Watts and Strogatz [8] showed that complex (biological and non-biological) networks could be described using only two parameters. The first is a measure of local functional interconnectedness, called the clustering coefficient C. The second, termed path length (L), describes global functional connectivity of the network (Figure 1). These parameters are computed for all nodes in the network and subsequently averaged to determine the functional connectivity of the overall network. Networks can range between highly ordered networks (high C and a high L) and random networks (low C and a low L). It has been suggested that a small world network, which is in between an ordered and a random network, is optimal for synchronizing neural activity between brain regions [9–11]. Especially electroencephalography (EEG) and magnetoencephalography (MEG) are methods well-suited for Graph Theory-based investigations of the human brain, owing to their high time resolution that allows to capture ongoing brain dynamics. Using Graph Theoretical analysis, Ferri et al. [12] have shown that the functional connectivity of the brain as measured using EEG during sleep becomes more similar to the organization of a small-world network for frequencies < 15 Hz. In addition, we have shown [4] that after sleep deprivation, the overall brain network is more random as compared to after sleep. Both studies support the idea that sleep is required to optimize the brain functional connectivity for processing information the following day [1, 13, 14]. It remains incompletely understood, however, whether sleep facilitates optimal functional connectivity equally across different brain areas: regional sensitivity of the functional brain network to sleep deprivation may underlie specific consequences for cognitive functioning. We acquired high-density EEG in a within-subject study of the effects of sleep vs. sleep deprivation on subsequent waking brain activity (Figure 2). We adapted Graph theoretical analysis to compute C and L for each node in the network individually and applied a cluster analysis to determine regional changes in functional connectivity. To our knowledge, this is the first study to assess topographical changes in functional connectivity at the individual electrode level after sleep deprivation using Graph Theory.

## Results and discussion

### Graph analysis

**Figure 3:**
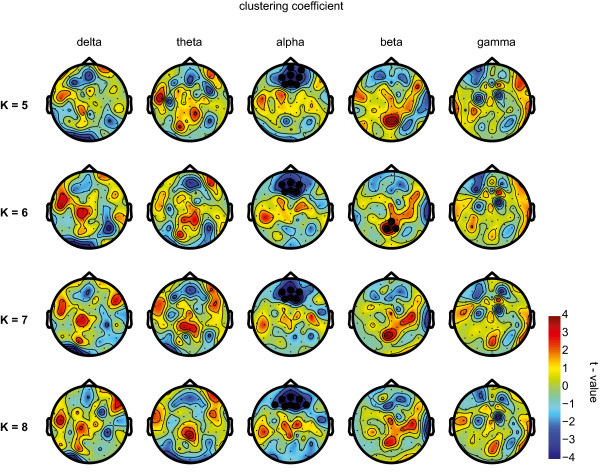
**Local integration.** The difference in distribution of clustering coefficient (C) during resting wakefulness following sleep deprivation as compared to wakefulness following a normal night of sleep. For all frequencies, the topographies show the uncorrected t-values plotted on the scalp for different values of K (5, 6, 7, and 8). Thick black dots indicate electrodes belonging to significant clusters (Monte-Carlo *P*-value < 0.001) in the eyes closed resting state. Note that there is a consistent frontal significant decrease in C in the alpha frequency band.

**Figure 4:**
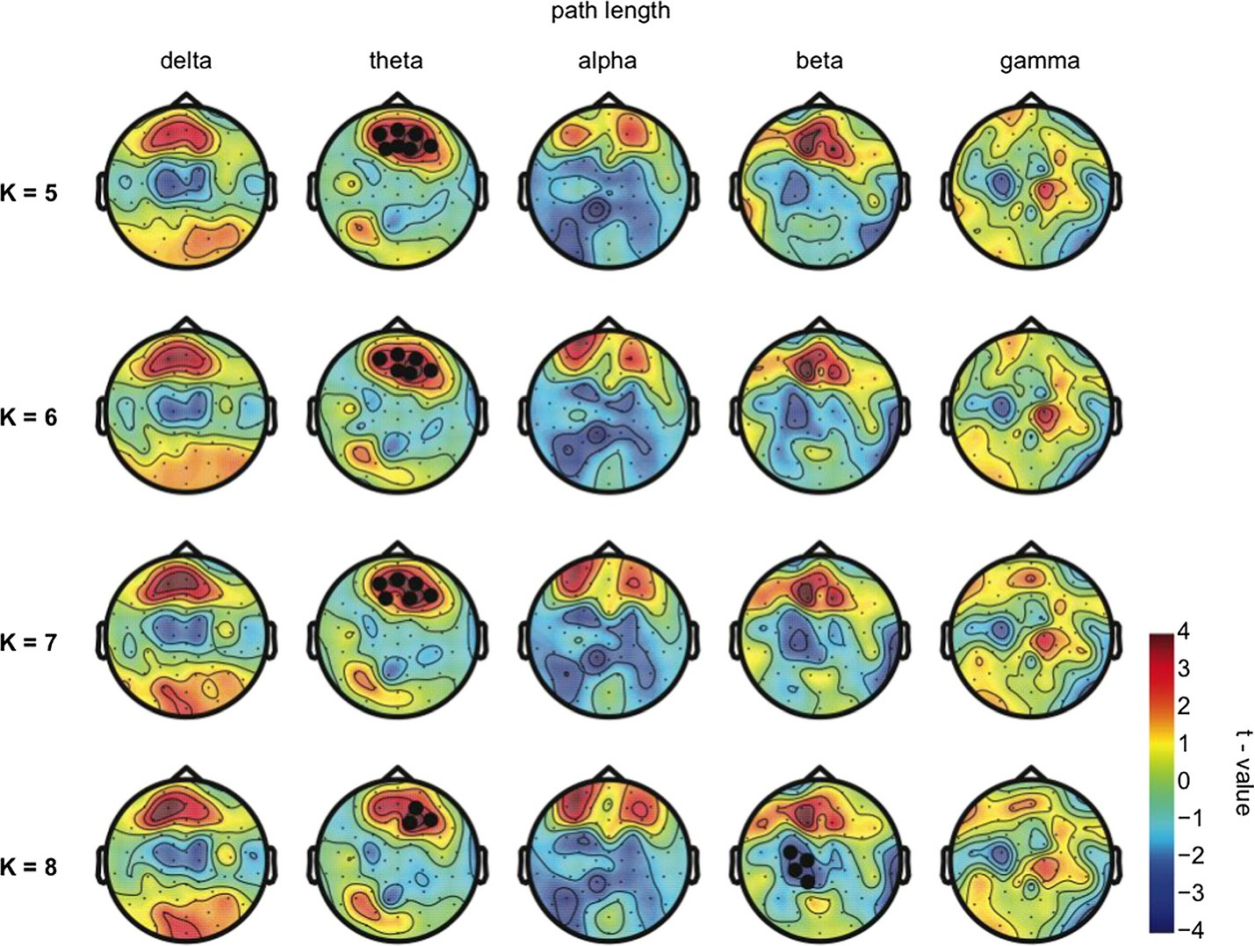
**Global integration.** The difference in distribution of path length (L) during resting wakefulness following sleep deprivation as compared to wakefulness following a normal night of sleep. For all frequencies, the topographies show the uncorrected t-values plotted on the scalp for different values of K (5, 6, 7, and 8). Thick black dots indicate electrodes belonging to significant clusters (Monte-Carlo *P*-value < 0.02) in the eyes closed resting state. Note that there is a consistent frontal significant increase in L in the theta frequency band.

In the eyes-closed condition, the distribution of the sleep deprivation-induced changes (*t*-values) in cluster coefficient C and path length L was robust across different values of degree K (Figures 3 and 4). A significant decrease in C was exclusively found at a prefrontal location in the alpha frequency band (*P*-value = 0.001 for all values of degree K, Figure 3). L was significantly increased frontally only in the theta frequency band (*P*-value < 0.02 for all values of degree K, Figure 4). These significant changes were found regardless of K (i.e. 5, 6, 7, or 8). C was significantly increased posteriorly in the beta frequency range (*P* = 0.001), but only for K = 6. L was decreased centrally in the beta range only for K = 8 (*P* = 0.003). No other significant changes were detected in the delta, beta or gamma frequency bands (all *P*-values >0.05). Sleep deprivation did not significantly affect connectivity parameters in any of the frequency bands (i.e. delta, theta, alpha beta and gamma) during in the eyes-open condition (all *P*-values > 0.05).

### Volume conduction effects

Mediation analysis was applied to evaluate whether the regional changes in alpha and theta connectivity (cluster coefficient I and path length (L), respectively) could be secondary to regional changes in alpha and theta power. The Sobel test of Mediation was done for all electrodes within the significant clusters for K = 6 (representative for the other values of K). Power spectral density was computed for these electrodes and entered as a mediator variable in the mediation regression models, as described in the method section. A sleep deprivation-induced decrease in alpha power partially mediated sleep deprivation-induced changes in alpha connectivity (β without PSD = −0.075 (*t* = −4.94, *P* < 0.001), β with PSD −0.057 (*t* = −3.48, *P* < 0.001), Sobel test *Z*-score = −3.22 (*P* < 0.01)). This mediation effect was only partial since sleep deprivation remained a significant predictor of the cluster coefficient in the alpha frequency band in a multiple regression model that included both power and sleep deprivation as regressors to predict connectivity parameters. A sleep deprivation-induced increase in theta power did not mediate sleep deprivation-induced changes in theta connectivity since adding PSD to the regression model with sleep deprivation as a predictor of connectivity parameter L did not diminish its effect on theta connectivity (β without PSD = 0.105 (*t* = 3.53, *P* < 0.001), β with PSD 0.125 (*t* = 4.09, *P* < 0.001)).

### Main findings and implications

Our findings indicate that total sleep deprivation alters brain functional connectivity in a topographically specific way. Sleep deprivation most prominently affected functional connectivity involving electrodes overlying prefrontal areas. In the alpha frequency band cluster coefficient C, a measure of local functional interconnectedness, and in the theta frequency band path length (L), a measure of global functional connectivity, were significantly decreased and increased, respectively, in a cluster overlying the prefrontal cortex. This was consistent across different degree levels (K = 5, 6, 7, or 8). The changes in path length for the theta frequency band were not secondary to changes in power, making it unlikely that this result was caused by volume conduction. The changes in the cluster coefficient in the alpha frequency band are at least partially independent of changes in power. Interestingly, our results indicate that the effects of sleep deprivation on the contribution of frontal areas to the brain network differ depending on frequency band: for interactions in the alpha frequency range, the network showed regionally reduced clustering; in addition, interactions in the theta frequency range showed that the network was characterized by higher path length after sleep deprivation. Our findings are in agreement with fMRI studies showing an imbalance in the activation of posterior versus anterior brain regions [1, 15] of the default mode network (DMN) after sleep deprivation. It should be noted that these studies derived DMN activity from task related deactivation, rather than examining it during a resting state. Even though this is an important difference with our study, together these results point out that prefrontal brain regions are affected by sleep deprivation. An advantage of our method, in comparison with fMRI based studies, is the possibility of detecting more subtle and differential changes depending on frequency band; indeed, we show different results in the alpha and theta frequency band, that both indicate reductions in local and global efficacy, respectively, of the frontal regions as part of the network.

Previous research [16] has shown that the medial prefrontal cortex is one of the hubs in the DMN to which all other parts of the DMN are correlated. The finding of the current study shows parallels with studies revealing that especially prefrontal functions such as working memory [2], inhibition [17] and emotion regulation [18] suffer from sleep deprivation. This raises the question whether switching between the intrinsically driven ‘resting’ state and the extrinsically driven ‘active’ state is mediated by the prefrontal cortex and whether this mediation in part depends on the effects of sleep. Although we cannot infer this based on our results, previous studies [19, 20] suggest that this is indeed the case, showing that a network based on the anterior prefrontal cortex, the so-called ‘fronto-parietal control network’, acts as a mediator between two other brain networks, i.e. the default mode brain network and competing networks supporting externally driven cognition (e.g. the dorsal attention network).

Significant differences in prefrontal functional connectivity after sleep deprivation relative to NS were selectively visible in the alpha and theta frequency band. This is partly in agreement with the study by Koenis et al. [4] who showed that global network properties, defined by graph theory, in the alpha, theta and gamma band moved to a more random network after sleep deprivation compared to after sleep. Note that while Koenis et al. [4] found an overall decrease in path length in the theta frequency band, in this study we found an increase in path length in electrodes covering prefrontal locations. The difference between these results is due to the fact that, in case of the study of Koenis et al. [4], a local increase of a graph theoretical parameter will remain undetected when there is an overall decrease in the same graph theoretical parameter. This implies that studying topographical changes in connectivity in addition to global connectivity offers a more refined view of changes in network properties.

Previous research has shown that alpha and theta power in the wake EEG are decreased and increased, respectively, after sleep deprivation [21–23]. Moreover, power in these frequency bands correlated with activity of the fMRI DMN [24–26]. Instead of EEG power at electrode level, which reflects locally synchronized neural activity, we studied synchronization between EEG electrodes, representing interactions between underlying sources, composed of groups of neurons. We found significant changes in the eyes closed condition only, but do not want to exclude the possibility of more subtle effects of sleep deprivation on functional connectivity in the eyes open condition that did not reach significance in our study.

We can only speculate which aspect of sleep plays a role in maintenance of functional connectivity of the prefrontal cortex since we did not record sleep EEG of the subjects in our experiment. The most likely candidate is NREM sleep since it seems to have a restorative effect on prefrontal areas [27]. For example, cerebral blood flow is particularly low in this area during NREM sleep [28]. Furthermore, a study by Mander et al. [29] showed that an increase in delta band power during recovery sleep (after a night of sleep deprivation) improved inhibitory performance during a Go/NoGo task through an effect on the prefrontal cortex. The restorative function of slow wave sleep may not be limited to task-related brain activity [29, 30] but may also be of importance for resting state activity; the occurrence of slow waves has been related to brain regions comprising parts of the default mode network [30, 31]. Further support for a beneficial effect of especially NREM sleep on prefrontal functioning is given by the findings that boosting of prefrontal slow waves by means of transcranial direct current stimulation (TDCs) improves declarative memory, while suppressing them interferes with brain activation necessary for proper memory formation [32].

Further studies are needed to evaluate the intriguing possibility that NREM sleep is of importance in maintaining proper connectivity of the prefrontal cortex within the resting state network, and in maintaining the ability for fast ‘switching’ between intrinsically and extrinsically driven brain states.

### Limitations

Regarding the small world network parameters, only an unweighted C and L (i.e. based on a binary matrix instead of the absolute SL values) was computed. Because network parameters change depending on the threshold used for calculating C and L, it is difficult to determine which threshold K would lead to the ‘true’ representation of the network. In this study, however, different values of K were used as an attempt to overcome this problem and we only considered results robust when they appeared irrespective of K.

A further note of caution concerns the age range of the study group: as the age of participants spanned from 20–24 years of age, the findings represent those of healthy young brains and do not necessarily extrapolate to those of younger or older age groups.

## Conclusion

**Figure 5:**
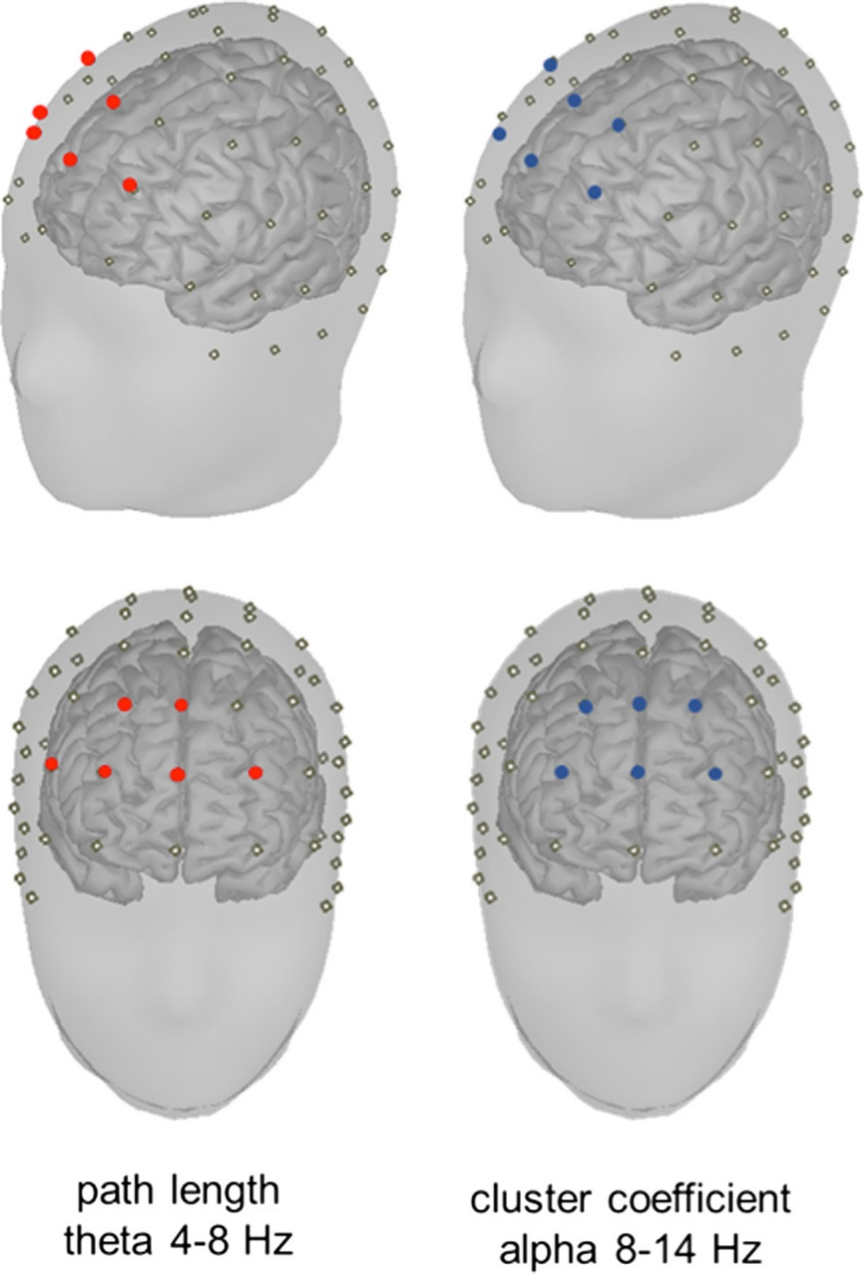
**Location of the electrodes showing significant changes in graph parameters.** Significant electrodes are plotted on the scalp for a representative value of K (K = 6). Only for nodes overlying the prefrontal cortex, sleep deprivation significantly attenuates the clustering coefficient in the alpha band and increases path length in the theta band.

In summary, this study showed that total sleep deprivation changes the structure of neural networks in prefrontal brain regions during the eyes closed resting state, as measured with EEG, possibly resulting in a network that is driven towards a less optimal state for information processing (Figure 5). Furthermore, we have shown that graph theory can be adapted to study local changes in network characteristics in addition to its usual application of quantifying global network properties only. One function of sleep may be to homeostatically regulate connectivity of especially prefrontal brain networks, allowing for optimal cognitive performance.

## Methods

### Participants

Eight healthy volunteers (5 males, age ± sd; 22 ± 1.77 yrs) participated in the study. All participants met the criteria of no self-reported: (1) sleep complaints (2) smoking (3) use of medication, including hormonal contraceptives (4) neurological or psychiatric disorders. Participants were asked to avoid consumption of beverages that contain caffeine or alcohol 12 hours prior to the experiment and during the experiment. All female subjects participated between day 4 and 12 of their menstrual cycle (follicular phase). All participants were instructed to keep a regular sleeping pattern the week prior to the experiment. This was verified with a sleep diary and actigraphy (Actiwatch, Cambridge Neuro-Technology Ltd., Cambridge, UK). Participants had to refrain from eating at least four hours before arrival at the sleep laboratory. The study protocol was approved by the medical ethics committee of the academic medical centre of the University of Amsterdam according to the declaration of Helsinki. Participants gave their written informed consent and received compensation for their participation in the experiment.

### Procedure

The experiment described in this article was part of a larger experiment which involved EEG measurements during a series of visuo-motor computer tasks following resting-state EEG measurements that are reported elsewhere [33]. Only relevant information about the procedure leading to this article will be described (Figure 2). The experiment consisted of two days with a minimum interval of two nights of normal sleep (mean interval ± std: 5.1 ± 4.7 nights). Prior to each day of the experiment, the participants had either a night of normal sleep (NS) or a night of total sleep deprivation (TSD) at their own homes. Compliance with the protocol was verified with actigraphy and a sleep diary. The order of the conditions was randomized and counterbalanced across subjects. On the day of the experiment, participants reported to the sleep laboratory at 8.30 h where they were prepared for EEG measurements. Resting state EEG was recorded during five sessions at 10.30 h, 12.00 h, 13.30 h, 15.00 h, and 16.30 h. Between the sessions participants had an isocaloric meal (230 kcal) and a non-caffeinated drink. EEG resting-state measurements were conducted during 4 minutes of alternating 1-minute epochs of eyes open and eyes closed. During these measurements subjects were sitting. They opened and closed their eyes upon a beeping sound. EEG was carefully monitored online for signs of sleep (slow rolling eye movements, sleep spindles and/or K-complexes). In case of such a sign subjects were woken up immediately.

### EEG acquisition

EEG data were collected using a 61 channel EEG cap (M10 Equidistant 61-Channel-Arrangement, Easycap, Herrsching, Germany) connected to a Micromed systemPlus recorder (version 1.04.0, Micromed, Treviso, Italy). An online high-pass filter of 0.015 Hz was applied. EEG data were digitized at a sampling rate of 1024 Hz with a digitizer sensitivity of 16 bits for ± 3.2 volts.

### Offline EEG preprocessing

Off-line EEG analysis was done using EEGLAB (v2008b) [34] and Fieldtrip [35] in MatLab 7.6 (The MathWorks, Natick, MA). Data were down sampled from 1024 Hz to 512 Hz and bad channels were interpolated, using the triangle-based linear interpolation method in MatLab. Movement artifacts were removed and independent component analysis (ICA) was applied to remove eye artifacts [36]. EEG recordings were re-referenced from common reference at Cz to average reference.

### Graph analysis

In this study the construction of connected graphs is based on the Synchronization Likelihood (SL) measure [37] as reported before [38, 39]. For each subject eight epochs (the data of all subjects contained at least eight artifact free epochs) of eight seconds (4096 data points) for each of the five sessions during each experimental day (NS and TSD) were included for further analysis. EEG data were band-pass filtered (EEGLAB default FIR filter) in the following frequency bands: delta (1–4 Hz), theta (4–8 Hz), alpha (8–13 Hz), beta (13–30 Hz), and gamma (30–45 Hz). Correlations between all channel combinations were calculated using SL. SL is sensitive to both linear and non-linear synchronization between two time series. To calculate SL, time-delay embedding vectors are constructed that represent the dynamical states of neuronal signals. Then, the times of recurrence of these states is assessed, followed by the calculation of the likelihood that the recurrent state of one signal is accompanied by the recurrent state of another signal (for a detailed description of SL see [37]). SL varies between a reference value *p*_*ref*_*,* the likelihood of a coincident pattern recurrence in case of independent time series and 1 in case of totally dependent time series. For this study, *p*_*ref*_ was set to 0.01. The other parameters for the computation of the SL were specified in accordance with Montez et al. [40]. From the (61 x 61) SL matrix a binary matrix was calculated by applying a threshold θ such that the average number of connections (degree K) per electrode (node) was fixed. This was done for different values of degree K (K =5, 6, 7 or 8) to investigate the robustness of the results. The rationale for keeping average degree K low is to make sure only the strongest connections will remain. A very high K would lead to the inclusion of weaker, possibly spurious, connections.

Next, the binary matrix was used for calculation of graph characteristics clustering coefficient C and path length L. C can take a value between 0 and 1 and represents the proportion of neighbors (i.e. a node that is directly connected to another node) of a node that are connected to each other. L is computed as the average number of steps that have to be taken from one node to any other node in the network, taking the shortest route. Because in this analysis unconnected nodes were unavoidable, we assigned ∞ to unconnected nodes as proposed by Newman [41] before calculating the harmonic mean over all eight epochs for every session and condition. In order to detect major changes after TSD, both C and L were averaged over all 5 sessions for the NS and TSD condition using the harmonic mean. SL and graph parameters were computed using a program created by one of the authors (DS).

### Statistical analyses

Because we were interested in topographical changes in C and L and did not want to make a priori assumptions about the location of changes in these parameters, we analyzed the network measure for each electrode individually. To correct for multiple comparisons, we used a non-parametric cluster permutation analysis as described by Maris and Oostenveld [42]. This analysis is suitable for the analysis of high density EEG data as it deals with the multiple comparisons problem by applying a cluster correction on the data. The following steps were taken to assess local significant changes in C and L. A pair wise *t-*test was done between NS and TSD for every node. Nodes with a *P*-value < 0.05 and at least two significant neighboring nodes were considered part of a cluster. Test-statistics for the cluster permutation test were based on the cluster with the largest (absolute) summed *t*-values. A number of 1000 permutations was used for calculation of the Monte Carlo *P*-value. Local changes between conditions were considered significant if the Monte Carlo *P*-value was smaller than the critical alpha-level of 0.025 (two sided test).

### Volume conduction effects

Volume conduction is a serious confounder in EEG connectivity studies [43]. High synchronization values between electrodes could be caused by picking up activity from the same, i.e. common sources.

We applied a Sobel test of mediation [44] to address this problem. A variable, in this case power spectral density (PSD) within one of the previously found significant clusters of C and L in one of the selected frequency bands, is considered a mediator when (1) the independent factor (condition NS/TSD) significantly affects the mediator (PSD within a significant cluster of C or L) (2) the independent factor significantly affects the outcome variable (C or L) (3) the mediator significantly affects the outcome variable (4) the effect of the independent factor disappears or is diminished when adding the mediator to the model with the independent factor predicting the outcome variable. The mentioned criteria are tested with mixed effect regression models with subjects nested within day and electrode nested within subjects as random factors. All regression models were done with the package lme4 [45] in R [46]. Only electrodes within the found significant clusters for C and L were used for this analysis. If the above criteria are met, significance of the mediator is determined by calculating the Sobel test *Z-*score using the formula a*b/SQRT(*b*^2^*s_a_^2^ + *a*^2^*s_b_^2^) where a = β of model 1, b = β of model 2; *s*_a_ and *s*_b_ are the standard errors of models 1 and 2, respectively. For this analysis, the power spectrum of the relevant clusters were obtained using a fast Fourier transform after application of Welch’s averaged method [47] with a (two seconds) Hamming window using Fieldtrip [35].

### Visualization

Figure 5 was created using Brainstorm (Tadel et al. [48]) (which is documented and freely available for download online under the GNU general public license: http://neuroimage.usc.edu/brainstorm). EEG Electrode positions were transformed to fit the default anatomy in Brainstorm, Colin27, an MNI brain with a 1 mm resolution.
